# Retraction Note: Integral effects of brassinosteroids and timber waste biochar enhances the drought tolerance capacity of wheat plant

**DOI:** 10.1038/s41598-025-00575-5

**Published:** 2025-05-06

**Authors:** Irfana Lalarukh, Syeda F. Amjad, Nida Mansoora, Sami A. Al-Dhumri, Abdullah H. Alshahri, Mohammad M. Almutari, Fatimah S. Alhusayni, Wasimah B. Al-Shammari, Peter Poczai, Mohamed H. H. Abbas, Doaa Elghareeb, Khadija tul Kubra, Ahmed A. Abdelhafez

**Affiliations:** 1 Department of Botany, Government College Women University Faisalabad, Faisalabad, 38000 Pakistan; 2 Department of Botany, University of Agriculture Faisalabad, Faisalabad, 38000 Pakistan; 3 Department of Biology, Al Khurma University College, Taif University, P.O. Box 11099, 21944 Taif, Saudi Arabia; 4 Civil Engineering Department, College of Engineering, Taif University, P.O. Box 888, 21974 Taif, Saudi Arabia; 5 Ministry of Environment, Water and Agriculture, 11442 Riyadh, Saudi Arabia; 6 Ministry of Environment, Water and Agriculture, Saudi Arabia, Ministry of Environment, P.O. Box 7878, 11195 Riyadh, Saudi Arabia; 7 Department of Biology, College of Science, University of Hail, P.O. Box 2440, 55476 Hail, Saudi Arabia; 8 Botany Unit, Finnish Museum of Natural History, University of Helsinki, 00014 Helsinki, Finland; 9 Department of Soils and Water, Faculty of Agriculture, Benha University, Benha, Egypt; 10 Department of Biology, Jumum College University, Umm Al-Qura University, P.O Box 7388, 21955 Makkah, Saudi Arabia; 11 Agriculture Genetic Engineering Research Institute (AGERI), Agriculture Research Centre, Giza, Egypt; 12 Department of Agronomy, Faculty of Sciences, University of Agriculture Faisalabad, Faisalabad, Pakistan; 13 Department of Soils and Water, Faculty of Agriculture, New Valley University, Kharga, Egypt; 14 National Committee of Soil Sciences, Academy of Scientifc Research and Technology, Cairo, Egypt

Retraction of: *Scientific Reports* 10.1038/s41598-022-16866-0, published online 27 July 2022

The Editors have retracted this Article.

Following publication, the irrelevant citation of Vickers, 2017^1^ was brought to the attention of the Editors. Investigation by the Editors has uncovered serious authorship irregularities. Editors no longer have confidence in the reliability of this Article.

Ahmed A. Abdelhafez agrees with the retraction. Nida Mansoora disagrees with the retraction. Irfana Lalarukh and Peter Poczai did not explicitly state whether they agree or disagree with the retraction. Syeda F. Amjad, Sami A. Al-Dhumri, Abdullah H. Alshahri, Mohammad M. Almutari, Wasimah B. Al-Shammari, Mohamed H. H. Abbas, Doaa Elghareeb, and Khadija tul Kubra did not respond to the Editors’ correspondence about this retraction. Editors were not able to confirm current contact details for Fatimah S. Alhusayni.
